# Tobacco use and associated factors among adults reside in Arba Minch health and demographic surveillance site, southern Ethiopia: a cross-sectional study

**DOI:** 10.1186/s12889-021-10479-4

**Published:** 2021-03-04

**Authors:** Befikadu Tariku Gutema, Adefris Chuka, Gistane Ayele, Wubshet Estifaons, Zeleke Aschalew Melketsedik, Eshetu Zerihun Tariku, Zerihun Zerdo, Alazar Baharu, Nega Degefa Megersa

**Affiliations:** 1 School of Public Health, Arba Minch University, P.O.Box 21, Arba Minch, Ethiopia; 2 Arba Minch Health and Demographic Surveillance Site (HDSS), Arba Minch, Ethiopia; 3 CARE Ethiopia Hawassa Project Office, Hawassa, Ethiopia; 4 School of Nursing, Arba Minch University, Arba Minch, Ethiopia; 5 Department of Medical Laboratory Science, Arba Minch University, Arba Minch, Ethiopia; 6 Department of Computer Science, Arba Minch University, Arba Minch, Ethiopia

**Keywords:** Tobacco, Smoked, Smokeless, Health and demographic surveillance site (HDSS), Ethiopia

## Abstract

**Background:**

Tobacco use is one of the world-leading preventable killers. There was a varied prevalence of tobacco use and cigarette smoking across different areas. The aim of the study was to assess the prevalence and factors associated with current tobacco use among adults residing in Arba Minch health and demographic surveillance site (HDSS).

**Methods:**

A community-based cross-sectional study was conducted among adults residing in Arba Minch HDSS in 2017. The estimated sample size was 3368 individuals which were selected by simple random sampling techniques using Arba Minch HDSS dataset. Data collection tools were obtained from the WHO STEPwise. Current use of tobacco, which defined as the current use of smoked and/or smokeless tobacco, was considered as the dependent variable. A binary logistic regression model was used to identify candidate variables for the multivariable logistic regression model. An adjusted odds ratio (AOR) at a *p*-value of less than 0.05 was used to determine a statistically significant association between independent and dependent variables.

**Result:**

The prevalence of tobacco use among adults was 20.2% (95% CI: 18.9–21.6%). The current use of smoked and smokeless tobacco were 17.1% (95%CI: 15.8–18.4%) and 9.7% (95%CI: 8.8–10.8%), respectively. The current use of tobacco was significantly associated with sex (female [AOR 0.54; 95%CI: 0.42–0.68] compared to men), age group (35–44 [AOR 1.57; 95%CI: 1.14–2.17], 45–54 [AOR 1.99; 95%CI: 1.45–2.74], and 55–64 [AOR 3.26; 95%CI: 2.37–4.48] years old compared to 25–35 years old), physical activity (moderate physical activity level [AOR 0.65; 95%CI: 0.44–0.96] compared with low) and residency (highland [AOR 4.39; 95% CI: 3.21–6.01] compared with at lowlander). Also, heavy alcohol consumption (AOR 3.97; 95% CI: 3.07–5.12), and Khat chewing (AOR 3.07(95%CI: 1.64–5.77) were also associated with the use of tobacco among the study participants.

**Conclusion:**

Nearly one in five adults used tobacco currently in the study area, which is more than the national reports. Interventions for the reduction of tobacco use need to give due attention to men, older adults, uneducated, poor, and highlanders.

## Background

Tobacco use is one of the world-leading preventable killers, driving an epidemic of cancer, heart disease, stroke, chronic lung disease, and other non-communicable diseases (NCDs). In addition, it is a major preventable cause of premature death and disease worldwide [1, 2]. Globally in 2017, around 1.4 billion people aged 15 years or older use tobacco (1.07 billion smokers and 367 million use smokeless tobacco) and the age-standardized global average prevalence of smoking is 19.2% [3]. It caused around 8 million deaths a year, with a substantially elevated risk of mortality among adults aged above 45 years [3, 4]. Evidences have shown that in the poorest households in some low-income countries as much as 10% of total household expenditure is on tobacco [5, 6]. Tobacco use is an important contributing factor to the global burden of morbidity and mortality [7].

Tobacco use increases the risk of death from many diseases, including ischemic heart disease, cancer, stroke, chronic obstructive pulmonary disease, diabetes, and other fatal and non-fatal diseases [8]. Based on the 2015 mortality burden in Ethiopia, ischemic heart disease and tuberculous are among the leading top five causes of death, which are also tobacco-related diseases [9]. In addition, these diseases (including cardiovascular diseases) were also from the leading top five disability-adjusted life years in the same year [9]. Smoking prevalence projections for 2025 showed that the prevalence in African will increase to 18% [10]. In sub-Saharan Africa, smoking prevalence varied widely even between similar countries. Smoking rates are higher among countries in the eastern and southern regions of Africa [11]. In addition to its direct morbidity and mortality on the users, second-hand smoke contributes to more than half-billion death worldwide [12]. According to the World Health Organization (WHO) report, the estimated current tobacco use in Ethiopia is 3.9% (7.6 and 0.3% for males and females) in the year 2020 [10]. Reports showed that the prevalence of tobacco use in Ethiopia ranges from 4.1 to 28.0% [13–16]. For instance, a school-based study at Hawassa and Jimma towns of Ethiopia showed that current cigarette smoking among adolescents was 17.2% [17]. Also, the daily use of smokeless tobacco was 33.1% among adult pastoralists in the Borena zone, Ethiopia [18]. Contrarily, in Aleta Wondo, none of the women who participated in the study reported current tobacco use [19]. These reports showed that the prevalence of tobacco use in Ethiopia is varied significantly. However, the country is at risk of the tobacco epidemic given the shift in the tobacco industry towards targeting low- and middle-income countries, particularly Africa, to recruit new users [5].

In 2015, by considering the health and economic effect of tobacco use, Ethiopia also implements the WHO MPOWER policy technical package under the WHO Framework Convention on Tobacco Control [5, 20]**.** As a result, Ethiopia has made progress on tobacco control in recent years. The Ethiopian parliament passed bills in 2012 and 2019. Under the proclamation of No.759/2012, the advertisement of cigarettes or other tobacco products was prohibited. On top of this, in 2019 (Proclamation No. 1112/2019) the Parliament passed additional law which prohibits smoking and tobacco use in public places, the sale of tobacco products for age less than 21 years, and taxation on tobacco product. However, people continue to die and become sick needlessly, and the costs to society from tobacco use continue to mount [21]. To make the proven tobacco control tools work for citizens’ wellbeing, periodic monitoring of tobacco use is important to enrich the country with quality data. In addition, local data is needed to inform proactive contextualized tobacco control intervention [22]. For the prevention of NCDs, identifying the major risk factors is important in assisting the planning, determining public health priorities, and monitoring the long-term interventions. The WHO STEPwise approach to Surveillance (STEPS) of NCDs risk factors is based on a sequential level of surveillance of different aspects of NCDs. It is a method using standardized tools to ensure comparability over time and across locations [23]. For overtime comparability of the surveillance data, Health and demographic surveillance site (HDSS) is considered as an appropriate platform. HDSS is a site of filed for longitudinal follow-up of well-defined primary subjects (individuals, households, and residential units) and related demographic characteristics of a population living in a well-defined geographic area [24]. Hence, the aim of the study was to assess the prevalence and factors associated with current tobacco use among adults residing in Arba Minch HDSS, which was collected based on WHO STEPS approach.

## Methods

### Study design and setting

A cross-sectional study was conducted to assess the prevalence and factors associated with tobacco use among adults residing in Arba Minch HDSS. Arba Minch HDSS operates in 9 of 29 Kebeles of Arba Minch Zuria District, Gamo Zone of Southern Ethiopia. The administrative town of the district, Arba Minch, found 505 Km South of Addis Ababa. The data was collected from April to June 2017.

### Study participants

The study was conducted among adult population age between 25 and 64 years, who were residing in Arba Minch HDSS. Pregnancy and history of recent delivery (up to 8 weeks) were the exclusion criteria. The sample size was estimated using single population proportion formula with the consideration of one of the risk factors for NCDs (prevalence of hypertension), which was 22.4% [25], 95% confidence level (CI), and 5% margin of error. In addition, 1.5 design effect was considered, and the estimated sample size was 396. Five percent non-response rate and based on the WHO STEPS guideline for sample size calculation, eight groups were considered to have an adequate level of precision for every age-sex estimate [26]. Then the estimated final sample size was 3368. Using the Arba Minch HDSS dataset, a simple random sampling technique was followed to select the study participants.

### Data collection tools and procedure

Tools for collecting socio-demographic information, tobacco and alcohol use, food intake and physical activity were obtained from the WHO STEPwise [26]. The wealth index of the participants was determined based on the ownership of the productive asset and characteristics of the house. Mental stress was assessed using a self-reporting questionnaire (SRQ-20) [27, 28]. Data was collected by Arba Minch HDSS filed workers. Training was given for three days for both data collectors and supervisors. A pre-test was conducted on 2% (on 68 individuals) of the sample size. Details of its methods have been described elsewhere [29–31].

### Variables

The independent variables include socio-demographic status, physical activity level, and alcohol consumption. Socio-demographic variables included sex, age, residency, religion, ethnicity, marital status, occupational status, educational level, wealth status and altitude. Using the principal component factor analysis, the wealth index was computed and grouped into five [28]. Altitude blow 1800 m, between 1800 to 2400 m, above 2400 m classified as lowland, midland, and highland, respectively [32]. Using a self-reporting questionnaire (SRQ-20), those scored from 0 to 4, 5–12, and above 12 were considered as mild, moderate, and severe mental stress, respectively [27]. The index for questions from the STEPS tool was generated in accordance with the manual. Heavy alcohol consumption was generated based on the individual consumption of alcohol with six or more drinks for men, and four or more for women on a single occasion [33]. Physical activity levels was grouped with average metabolic equivalents-minutes per week below 600 as low, for 600–3000 as moderate and for above3000 as high activity level [26]. As an outcome variable, the current use of tobacco, which defined as the current use of smoked and/or smokeless tobacco, was considered. In addition, the current use of smoked and smokeless tobacco were analyzed separately.

### Data processing and analysis

Data was entered into EPI-data version 3.1 statistical software and analysis was done by STATA version 14. All covariates with a *p*-value of less than 0.25 in bivariate analysis were considered for a multivariable logistic regression. To assess the presence of an association between current tobacco use and independent variables, a p-value of less than 0.05 in the final model was considered statistically significant. Variable inflation factors were used to assess the presence of multicollinearity, and the maximum value was 1.71. The goodness-of-fit model was assessed using the Hosmer-Lemeshow test and the *P*-value for current use of (smoked and/or smokeless) tobacco was 0.653. In addition, the *P*-values for smoked and smokeless tobacco use were 0.112 and 0.067, respectively.

## Results

### Socio-demographic characteristics of the study participants

The total number of participants in the study was 3346 with a response rate of 99.3%. The mean (SD) age was 44.6(11.2) years with nearly equally distributed among age groups and sex. Rural residents (83.7%), Gamo ethnic (81.1%) and married (87.9%) were majority of the study participants. The most common occupation was farming which incorporates 53.2% of the participants. Regarding educational attainment, only 30.3% attended formal education, with the majority attended only primary education (Table 1).

**Table 1:** Socio-demographic characteristics of the study participants (3346) by current tobacco use in Arba Minch HDSS, Southern, Ethiopia

Variable	Categories	Total Participants	Non-Current User	Current Tobacco Use		
				Smoked & Smokeless	Smoked	Smokeless
		N (%)	N (%)	N (%)	N (%)	N (%)
Sex	Male	1674(50.0)	1235(46.3)	439(64.8)	368(64.4)	230(70.6)
	Female	1672(50.0)	1434(53.7)	238(35.2)	203(35.6)	96(29.4)
Age group	25–34	804(24.0)	727(27.2)	77(11.4)	46(8.1)	51(15.6)
	35–44	863(25.8)	710(26.6)	153(22.6)	120(21.0)	84(25.8)
	45–54	845(25.3)	656(24.6)	189(27.9)	163(28.5)	81(24.8)
	55–64	834(24.9)	576(21.6)	258(38.1)	242(42.4)	110(33.7)
Residency	Rural	2801(83.7)	2174(81.5)	627(92.6)	551(96.5)	294(90.2)
	Urban	545(16.3)	495(18.5)	50(7.4)	20(3.5)	32(9.8)
Marital status	Unmarried*	405(12.1)	354(13.3)	51(7.5)	41(7.2)	25(7.7)
	Married	2941(87.9)	2315(86.7)	626(92.5)	530(92.8)	301(92.3)
Ethnicity	Gamo	2713(81.1)	2088(78.2)	625(92.3)	546(95.6)	288(88.3)
	Zeysie	290(8.7)	273(10.2)	17(2.5)	6(1.1)	17(5.2)
	Other**	343(10.3)	308(11.5)	35(5.2)	19(3.3)	21(6.4)
Religion	Protestant	2104(62.9)	1992(74.6)	112(16.5)	53(9.3)	72(22.1)
	Orthodox	1054(31.5)	609(22.8)	445(65.7)	403(70.6)	245(75.2)
	Other	188(5.6)	68(2.5)	120(17.7)	115(20.1)	9(2.8)
Occupation	Farmer	1779(53.2)	1274(47.7)	505(74.6)	446(78.1)	253(77.6)
	Daily laborer	257(7.7)	225(8.4)	32(4.8)	22(3.9)	15(4.6)
	Merchant	146(4.4)	134(5.0)	12(1.8)	5(0.9)	7(2.1)
	Housewife	914(27.3)	812(30.4)	102(15.1)	80(14.0)	36(11.0)
	Employed at Org	106(3.2)	96(3.6)	10(1.5)	5(0.9)	6(1.8)
	Unemployed	119(3.6)	103(3.9)	16(2.4)	13(2.3)	9(2.8)
	Other	25(0.8)	25(0.9)	0(0.0)	0(0.0)	0(0.0)
Educational status	No formal education	2334(69.8)	1764(66.1)	570(84.2)	504(88.3)	266(81.6)
	Primary school	768(23.0)	676(25.3)	92(13.6)	60(10.5)	50(15.3)
	Secondary & above	244(7.3)	229(8.6)	15(2.2)	7(1.2)	10(3.1)
Wealth Index	1st quantile	1039(31.1)	868(32.5)	171(25.3)	136(23.8)	79(24.2)
	2nd quantile	690(20.6)	500(18.7)	190(28.1)	177(31.0)	75(23.0)
	3rd quantile	608(18.2)	483(18.1)	125(18.5)	109(19.1)	64(19.6)
	4th quantile	479(14.3)	357(13.4)	122(18.0)	102(17.9)	62(19.0)
	5th quantile	530(15.8)	461(17.3)	69(10.2)	47(8.2)	46(14.1)
Altitude	Lowland	1510(45.1)	1376(51.6)	134(19.8)	71(12.4)	86(26.4)
	Midland	427(12.8)	384(14.4)	43(6.4)	25(4.4)	30(9.2)
	Highland	1409(42.1)	909(34.1)	500(73.9)	475(83.2)	210(64.4)

*Unmarried: include single, divorced, separated and widow; ** Other include Goffa, Wolayta, Amhara

### Behavioral characteristics of the study participants

Majority (64.3%) of the study participants involved in high level physical activities. Heavy alcohol consumption was 11.5%. Nearly 2% of the study participates were chewed khat within 30 days and 4% had severe mental stress (Table 2).

**Table 2:** Behavioral characteristics of the of the study participants (*N* = 3346) by current tobacco use in Arba Minch HDSS, Southern, Ethiopia

Variable	Categories	Total Participants	Non-Current User	Current Tobacco Use		
				Smoked & Smokeless	Smoked	Smokeless
		N (%)	N (%)	N (%)	N (%)	N (%)
Heavy alcohol consumption	No	2962(88.5)	2473(92.7)	489(72.2)	413(72.3)	211(64.7)
	Yes	384(11.5)	196(7.3)	188(27.8)	158(27.7)	115(35.3)
Physical activity level	Low	791(23.6)	637(23.9)	154(22.8)	134(23.5)	73(22.4)
	Moderate	405(12.1)	358(13.4)	47(7.0)	31(5.4)	26(8.0)
	High	2150(64.3)	1674(62.7)	476(70.3)	406(71.1)	227(69.6)
Khat chewing within 30 days	Yes	66(2.0)	46(1.7)	20(3.0)	14(2.5)	12(3.7)
	No	3280(98.0)	2623(98.3)	657(97.0)	557(97.5)	314(96.3)
Mental stress	Mild	2219(66.3)	1790(67.1)	429(63.4)	356(62.3)	202(62.0)
	Moderate	995(29.7)	775(29.0)	220(32.5)	194(34.0)	106(32.5)
	Severe	132(4.0)	104(3.9)	28(4.1)	21(3.7)	18(5.5)

### Prevalence of tobacco use

Six hundred seventy-two (20.1%) and 382 (11.4%) study participants had a history of use of smoked and smokeless tobacco, respectively. In general, 784 (23.4%) study participants had a history of tobacco use. Six hundred seventy-seven (20.2%; 95% CI: 18.9–21.6%) adults were current tobacco users. Current use of smoked and smokeless tobacco were 17.1% (95%CI: 15.8–18.4%) and 9.7% (95%CI: 8.8–10.8%), respectively. From total study participants, 6.6% (95%CI: 5.8–7.5%) were currently used both smoked and smokeless tobacco.

Out of current smokers, 88.1% were smoking daily. Of current smokers, 441 (65.1%) recalled their age at smoking initiation. The mean (SD) age of smoking initiation was 21.8 (7.0) years. Related to the cessation of smoking, only 15.6% (89) of all smokers attempted to stop in the last 12 months. Three hundred twenty-six (9.7%) used smokeless tobacco. Of those who use smokeless tobacco, 178 (54.6%) use sniff tobacco and chewable tobacco together, and the remaining use only chewable tobacco. Of the smokeless tobacco users, 294 (90.2%) use it daily. From the total, 543 (16.2%) reported that there was someone in their home who smokes tobacco in the last 30 days.

### Factors associated with tobacco use

The factors found to be significantly associated with current tobacco use were sex, age, occupation, educational status, wealth status, alcohol use and Khat chewing, physical activity level, mental stress level, and altitude. Current tobacco use was significantly lower among females (AOR 0.54; 95%CI: 0.42–0.68). In a separate analysis, the prevalence of smoked and smokeless tobacco use among women had been nearly lower by 50% (AOR 0.56, 95% CI: 0.44–0.73 and AOR 0.54, 95% CI: 0.40–0.74, respectively) compared to men. The younger age group (25–35 years old) had significantly lower odds to use tobacco currently compared to older age groups (AOR for 35–44, 45–54, and 55–64 years old were 1.57 (95 CI: 1.14–2.17), 1.99(95% CI: 1.45–2.74), and 3.26 (95% CI: 2.37–4.48), respectively). The pattern is similar for smoked tobacco users. For smokeless tobacco use, the prevalence is only significantly higher for the age group 55–64 years (AOR 1.51; 95% CI: 1.02–2.23) compared with the younger age group (25–35 years old). There was significantly more tobacco use among farmers compared to other occupation (AOR 0.46; 95%CI: 0.31–0.68). It is also similar to smoked tobacco use. For smokeless tobacco use, housewives had significantly lower odds (AOR 0.58; 95%CI: 0.38–0.91) to being tobacco use compared with farmers. In addition, those participants with secondary and above (AOR 0.44; 95%CI: 0.21–0.93) education were lower odds to use smokeless tobacco currently compared with those with no formal education. Those from the fifth quantile wealth index household had to be lower odds (AOR 0.61; 95% CI: 0.41–0.92) to use smoked tobacco compared to the first quantile household. There is no difference among the wealth index groups for the current all form and smokeless tobacco use. Heavy alcohol consumption had nearly 4 times higher odds of being tobacco use (AOR of 3.97 (95% CI: 3.07–5.12), 3.55 (95% CI: 2.69–4.68), and 4.09 (95% CI: 3.10–5.39) for all form, smoked, and smokeless, respectively) compared to their counterpart. Khat chewers had higher odds to use tobacco than non-chewers in different forms (AOR for all form, smoked, and smokeless were 3.07(95%CI: 1.64–5.77), 3.64(95%CI: 1.73–7.66), and 2.22(95%CI: 1.07–4.59), respectively). The probability of use of tobacco reduces among those involved in moderate physical activities. This difference was significant for all form (AOR 0.65; 95% CI: 0.44–0.96), and smoked (AOR 0.49; 95% CI: 0.31–0.77) tobacco use. Those with severe mental stress had higher odds to use smokeless tobacco (AOR 1.79; 95% CI: 1.02–3.14) than mild. Among participants, those who were living at highland use tobacco more than those who live in lowland in all form (AOR 4.39; 95% CI: 3.21–6.01), smoked (AOR 6.79; 95% CI: 4.68–9.85), and smokeless (AOR 1.90; 95% CI: 1.28–2.81) (Table 3).

**Table 3:** Multivariable logistic regression analysis of factors associated with current tobacco use among study participants (*N* = 3346), Arba Minch HDSS, South Ethiopia

Categories	Current Tobacco Use					
	Smoked & Smokeless		Smoked		Smokeless	
	AOR (95% CI)	P-Value	AOR (95%CI)	P-Value	AOR (95% CI)	P-Value
Sex						
Male	Reference					
Female	0.54(0.42,0.68)*	< 0.01	0.56(0.44,0.73)*	< 0.01	0.54(0.40,0.74)*	< 0.01
Age group						
25–34	Reference					
35–44	1.57(1.14,2.17)*	0.006	1.97(1.34,2.9)*	0.001	1.19(0.81,1.75)	0.381
45–54	1.99(1.45,2.74)*	< 0.01	2.75(1.88,4.01)*	< 0.01	1.06(0.71,1.58)	0.759
55–64	3.26(2.37,4.48)*	< 0.01	5.07(3.48,7.40)*	< 0.01	1.51(1.02,2.23) *	0.038
Residency						
Rural	Reference					
Urban	1.26(0.86,1.85)	0.243	0.82(0.47,1.41)	0.462	1.23(0.77,1.97)	0.383
Marital status						
Unmarried	Reference					
Married	1.09(0.77,1.55)	0.624	1.04(0.70,1.55)	0.853	0.95(0.60,1.52)	0.839
Occupation						
Farmer	Reference					
Daily laborer	0.87(0.56,1.35)	0.537	0.88(0.52,1.48)	0.62	0.66(0.36,1.21)	0.178
Housewife	0.88(0.65,1.19)	0.407	0.83(0.60,1.16)	0.283	0.58(0.38,0.91)*	0.017
Other^@^	0.66(0.43,0.99)*	0.045	0.55(0.33,0.9)	0.019	0.65(0.39,1.09)	0.106
Educational status						
No formal education	Reference					
Primary school	0.94(0.70,1.27)	0.697	1.00(0.70,1.43)	0.994	0.72(0.49,1.04)	0.081
Secondary & above	0.56(0.30,1.04)	0.066	0.52(0.22,1.21)	0.129	0.44(0.21,0.93)*	0.032
Wealth Index						
1st quantile	Reference					
2nd quantile	1.02(0.78,1.34)	0.891	1.04(0.78,1.40)	0.773	0.99(0.69,1.42)	0.945
3rd quantile	0.91(0.68,1.22)	0.538	0.93(0.67,1.28)	0.637	1.16(0.80,1.68)	0.444
4th quantile	0.95(0.70,1.30)	0.765	0.84(0.60,1.18)	0.326	1.28(0.87,1.89)	0.207
5th quantile	0.72(0.51,1.01)	0.06	0.61(0.41,0.92)*	0.019	1.11(0.73,1.69)	0.629
Heavy alcohol consumption						
No	Reference					
Yes	3.97(3.07,5.12)*	< 0.01	3.55(2.69,4.68)*	< 0.01	4.09(3.10,5.39)*	< 0.01
Physical activity level						
Low	Reference					
Moderate	0.65(0.44,0.96)*	0.029	0.49(0.31,0.77)*	0.002	0.79(0.48,1.29)	0.344
High	0.91(0.72,1.16)	0.449	0.88(0.68,1.14)	0.324	0.89(0.65,1.2)	0.441
Khat chewing within 30 days						
No	Reference					
Yes	3.07(1.64,5.77)*	< 0.01	3.64(1.73,7.66)*	0.001	2.22(1.07,4.59)*	0.031
Mental stress						
Mild	Reference					
Moderate	1.11(0.90,1.37)	0.312	1.19(0.95,1.49)	0.132	1.12(0.86,1.45)	0.418
Severe	1.49(0.91,2.45)	0.115	1.44(0.81,2.55)	0.216	1.79(1.02,3.14)*	0.043
Altitude						
Lowland	Reference					
Midland	1.03(0.68,1.56)	0.903	0.96(0.57,1.62)	0.869	1.05(0.64,1.73)	0.843
Highland	4.39(3.21,6.01)*	< 0.01	6.79(4.68,9.85)*	< 0.01	1.9(1.28,2.81)*	0.001

* Significant at P-Value< 0.05

^@^ other include merchant, employee of the organizations, and unemployed

## Discussion

Our findings indicate that the prevalence of current tobacco use among the study participants was 20.2%. In addition, the prevalence of current smoked and smokeless tobacco use were 17.1 and 9.7%, respectively. The national prevalence of cigarette smoking was 3.5% based on the WHO report of 2017, 4.0% based on the national STEPS survey of 2015, 4.1% based on the Ethiopian DHS 2011 analysis, and 5.0% based on the Global Adult Tobacco Survey of Ethiopia 2016 [3, 14–16], which are lower as compared to this finding. In addition, the Ethiopia DHS report of 2016 showed that 4.3 and 0.8% of men and women, respectively, use tobacco [28]. In 2009, a survey using the WHO STEPS approach in southwest Ethiopia showed that the prevalence of all forms of tobacco use was 9.3% [34]. In general, tobacco use among the study population is higher compared to the national prevalence. A study conducted in eastern Ethiopia indicated that the proportion of current smokers was 28.6%, which is higher compared to this finding [13]. Nearly one third (33.1%) of pastoralists in the Borena zone, Ethiopia was daily use smokeless tobacco [18]. These significant differences might be related to the age group included in the study, and the difference in social norms and taboos regarding the use of tobacco. The global estimate of daily tobacco smoking was 15.2% in 2015 by Global Statistics on Addiction and 19.2% in 2017 by the WHO report on the Global Tobacco Epidemic [3, 7]. In addition, as it is also noted in a systematic review of tobacco smoking in sub-Saharan African countries, the smoking prevalence varies widely within the country [11]. This report also confirmed the significant variation in the prevalence of tobacco use in the country and this difference might be related to the difference in sociocultural norms.

In this study, the odds of all forms of tobacco use, smoked and smokeless tobacco use were significantly higher among men. This finding is similar to a study conducted in eastern Ethiopia, and national reports from Ethiopia including DHS (2011 and 2016), Global Adult Tobacco Survey (2016), NCD STEPS survey (2015) [13, 15, 16, 35]. A systematic review of tobacco smoking for Sub-Saharan African countries showed that the prevalence of tobacco use is more prevalent among men participants [11]. In addition, different studies showed that there is a higher prevalence of tobacco use among men compared to women [36–39]. The higher number of tobacco use among men might be linked with the feature of sex roles in society. Men have had greater social power, which may result in restriction or prohibition of tobacco use among women, which were the major cause of gender difference in tobacco use [40].

The likelihood of current tobacco use increases among older age groups compared to the young age group. This was similar to smoked tobacco use. Even if the odds of smokeless tobacco use increase with age, the only significant increase in odds was observed among the age group from 55 to 64 years. It is similar to the analysis done based on the Swiss Health Survey, which indicated that the proportion of smoking was increased among the older age groups [36]. Similarly, a report based on the analysis of the DHS datasets for 30 sub-Saharan African countries and a separate analysis for Ethiopia indicated that the prevalence of smoking significantly higher among the older age groups [35, 39]. This might relate to a delayed onset of tobacco use rather than the prevention of cigarette initiation [41]. A study conducted in Namibia indicated that the prevalence of smoking was higher among the age group of 20–29 years of age [42].

In this report, there was no significant difference in educational status regarding the use of tobacco, except for smokeless tobacco use. The odds of smokeless tobacco use significantly reduces with increasing educational levels. Consistent with the finding of this study, an analysis for 30 sub-Saharan African countries showed that the likelihood of smokeless tobacco use was higher among the uneducated group compared to the educated group. It also indicated that smoking had a weak relationship with education [39]. A study in Mumbai, India showed that the likelihood of smokeless tobacco use was inversely related to education level [43], which is similar to this finding. In addition, reports based on 2008–2010 Global Adult Tobacco Surveys indicated that the prevalence of tobacco use is higher among less educated [44, 45]. Even if it is not only for smokeless tobacco use, other studies also reported similarly that individuals with higher education were less likely to smoke tobacco [35, 36, 43].

Even if there was a gradient reduction in the odds of tobacco use across the wealth index in this study, there was no significant difference for all forms of tobacco use. However, the likelihood of smoked tobacco use was significantly lower among higher wealth index individuals (the fifth quantile compared to the first quantile). Analysis based on Ethiopian DHS of 2011 and 2016 indicated that smoking is significantly lower among individuals from higher household wealth [35]. Reports based on 2003 the World Health Survey of 48 Low- and Middle-Income Countries and Global Adult Tobacco Survey (based on 2008–2012) indicated that wealth status is a significant determinant of smoking risk. They showed that the prevalence of tobacco use is higher among low economic groups [44–46]. In addition to the high prevalence of smoking among individuals from lower socioeconomic, they smoke for longer durations than others [47]. The long duration of smoking may lead to experience financial stress and a deterioration of standards of living for longer periods of time. On top of the negative effect of tobacco use on health status, it will likely exacerbate existing health inequalities [5, 6, 47].

In this study, the odds of tobacco use was significantly higher among heavy episodic drinkers. Studies indicated that those who drink are very likely to smoke and these behaviors are highly correlated behaviors [48–52]. The use of heavy episodic drink of alcohol increases the probability of smoking and vice versa. In addition, both tobacco and alcohol use tend to be used together, for instance in times of stress [53].

Khat (*Catha edulis*) is a chewable fresh leave which contains amphetamine-like stimulatory and commonly used in East Africa [54, 55]. In this study, the odds of all forms of tobacco use and smoked and smokeless tobacco use were significantly higher among khat chewers. Different studies indicated that the proportion of tobacco use increase among khat users. Ethiopian NCD STEPS survey of 2015, and analysis based on Ethiopian DHS of 2011 and 2016, indicated that khat chewing is associated with tobacco use [15, 35]. In addition, a correctional study to see the correlation of concurrent khat and tobacco use in Yemen showed positive associations between khat and tobacco use [37]. A systematic review of the epidemiology of tobacco use and khat users concluded that the prevalence of tobacco use among khat users appears significantly higher [56]. It is expected that smoking helps to heighten the stimulant effect to khat and to break the negative psychological state [13] and the phenomena used to explain the association of alcohol and tobacco may be used for khat also [53].

The odds of all forms of tobacco and smoked tobacco use were significantly higher among those participants with moderate physical activity levels compared to lower physical activity levels. There is no significant difference in the use of smokeless tobacco. An analysis of the health survey of England indicated that individuals involving in sports activity were less likely to smoke. While distinguishing the type of physical activity by occupation or sport club-related physical activities, smoking was less common among sports-club members [57]. It is also indicated that smokers had low leisure-time physical activity [36].

In this study, the odds of smokeless tobacco use was significantly higher among severe mental stress participants compared to mild mental stress. Even if it is not significant, the odds of all forms of tobacco use increase with stress level. An experimental study indicated that stress significantly increases cigarette craving but did not increase smoking [58]. A study on smokeless tobacco and USA military deployment indicated that stress among the military associated with an increased risk of smokeless tobacco initiation [59]. A review of stress and tobacco use indicated a strong link between tobacco use and stress level. High-stress level predisposes for greater vulnerability for tobacco use [60].

The odds of tobacco use was significantly higher among those living in highland compared to lowlanders. This result is similar for smoked and smokeless tobacco use in a separate analysis. A study among Colombian Medical Schools showed that an increase in the prevalence of smoking with altitude [61]. A study based on a median altitude of state residents of the USA on chronic obstructive pulmonary disease mortality on a role for altitude showed that there is no association between altitude and smoking [62]. Increasing smoking prevalence at high altitudes is a serious public health issue since altitude aggravates at least some of the effects of smoking. An increase in the volume of red blood cells, a mechanism for coping with oxygen shortage at a higher altitude, was higher for non-smokers and respiratory function was for smokers who live in highland compared to lowland [63]. Opposite to the finding of this, an analysis based on Ethiopian DHS (2011 and 2016) indicated that the prevalence of tobacco smoking is significantly higher among lowlanders compared to highlanders [35]. The analysis based on Ethiopian DHS (2011 and 2016), noted that altitude information is missing for 47 community clusters.

We follow a standardized method and tool, which was described in the WHO STEPS guideline. This makes our finding easy to compare with studies conducted based on a similar method and definition of tobacco use. In this study, we consider three outcome variables of tobacco use for analysis, which are all form (smoked and/or smokeless), smoked and smokeless tobacco use, to see their association with independent variables. Because of the cross-sectional nature of the study, it is difficult to determine which comes first in variables like tobacco use and stress level, khat chewing, and heavy episodic drink of alcohol.

Tobacco use is significantly different in different parts of the country. The national and pocket study reports have a huge difference in the prevalence of tobacco use. The finding of this report is higher than the national reports. To uncover the differences in the use of tobacco, the national reports need to be disaggregated. On top of this, it is important to investigate the reason for the differences in the prevalence of tobacco use. In addition, the implementation of policies for combating tobacco use in Ethiopia needs to account for the local contexts of tobacco use prevalence. Further, the reason for the use of smokeless tobacco among severe mental stress level needs to be explored.

## Conclusions

Nearly one in five adults use tobacco currently in the study area, which is more than the national reports. In addition, nearly 17 and 10% use smoked and smokeless tobacco currently, respectively. Sex, age, heavy alcohol consumption, physical activity level, khat chewing and altitude of their residency were associated with tobacco use. Interventions for the reduction of tobacco use need to give due attention to men, older adults, uneducated, lower socio-economic status, and highlanders. Intervention on the reduction of heavy alcohol consumption and khat chewing may have an impact on tobacco use.

## Data Availability

The datasets used and/or analyzed during the current study are available from the corresponding author upon reasonable request.

## Abbreviations

DHS: Demographic and Health Survey
HDSS: Health and Demographic Surveillance Site
NCD: non-communicable disease
SRQ-20: Self-reporting questionnaire
STEPS: STEPwise approach to Surveillance
WHO: World Health Organization
